# Carbide Formation in Refractory Mo_15_Nb_20_Re_15_Ta_30_W_20_ Alloy under a Combined High-Pressure and High-Temperature Condition

**DOI:** 10.3390/e22070718

**Published:** 2020-06-28

**Authors:** Congyan Zhang, Uttam Bhandari, Congyuan Zeng, Huan Ding, Shengmin Guo, Jinyuan Yan, Shizhong Yang

**Affiliations:** 1 Department of Computer Science, Southern University and A&M College, Baton Rouge, LA 70813, USA; congyan_zhang@subr.edu (C.Z.); uttam_bhandari_00@subr.edu (U.B.); 2 Department of Mechanical and Industrial Engineering, Louisiana State University, Baton Rouge, LA 70803, USA; czeng8@lsu.edu (C.Z.); hding3@lsu.edu (H.D.); sguo2@lsu.edu (S.G.); 3 Lawrence Berkeley National Laboratory, Berkeley, CA 94720, USA; jyan@lbl.gov

**Keywords:** high-entropy alloy, compression deformation, synchrotron x-ray diffraction, diamond anvil cell, high-pressure and high-temperature property

## Abstract

In this work, the formation of carbide with the concertation of carbon at 0.1 at.% in refractory high-entropy alloy (RHEA) Mo_15_Nb_20_Re_15_Ta_30_W_20_ was studied under both ambient and high-pressure high-temperature conditions. The x-ray diffraction of dilute carbon (C)-doped RHEA under ambient pressure showed that the phases and lattice constant of RHEA were not influenced by the addition of 0.1 at.% C. In contrast, C-doped RHEA showed unexpected phase formation and transformation under combined high-pressure and high-temperature conditions by resistively employing the heated diamond anvil cell (DAC) technique. The new FCC_L1_2_ phase appeared at 6 GPa and 809 °C and preserved the ambient temperature and pressure. High-pressure and high-temperature promoted the formation of carbides Ta_3_C and Nb_3_C, which are stable and may further improve the mechanical performance of the dilute C-doped alloy Mo_15_Nb_20_Re_15_Ta_30_W_20_.

## 1. Introduction

High-entropy alloys (HEAs), which are considered as a new class of advanced metallic materials, are drawing increasing attention because of their promising properties [1,2,3], such as high strength at both room temperature and elevated temperatures [4,5], exceptional ductility [6], and toughness [7]. HEAs were initially defined as alloys containing five or more elements with equal or near-equal concentrations [8], while the later studies expended HEAs to include minor elements [9]. Many studies on HEAs were motivated by the possibility that properties may be tunable based on the elements contained in the HEAs. For example, Al-based HEAs [10,11,12,13] were studied for application on aircraft engines and nuclear reactors because of their low densities. On the other hand, because of the high melting temperature and high hardness of W and Re, HEAs that contained W or Re were expected to maintain advanced mechanical properties for high-temperature applications [5,14]. 

Beside the contained elements in HEA, doping with interstitial elements such as carbon to HEA has been announced to be an effective way to improve the mechanical properties [15,16,17,18,19,20,21,22]. The tensile yield strength of CoCrFeMnNi could increase from 371 MPa to 792 MPa just by adding 3.0 at.% C [22]. It was reported that the doped C atoms may have strong interactions with elements in HEA to form carbides, which usually lead to higher strength. As expected, both the yield strength and the ultimate tensile strength increase with the carbon content increasing in CoCrFeMnNi [22]. By analogy, the increase of both compressive strength and the plasticity of the Mo_0.5_NbHf_0.5_ZrTi refractory HEA (RHEA) was attributed to the formation of carbides (MC with M = Nb, Hf, Zr, and Ti), when the concertation of C was only 0.3 at.%, a dilute doping case [20].

The formation of carbides might be influenced by multiple aspects, like concentration of C, and atomic interactions between C and other elements in HEA. The pressure and temperature could also affect the formation of carbides. However, studying the interstitial C alloying HEAs under both high-pressure and high-temperature is a great challenge. The technique of synchrotron radiation, combined with double-sided laser heating or resistive heating, has become a unique method for combined high-temperature and high-pressure studies [23]. The laser-heated diamond anvil cell (LHDAC) [24,25,26] has become an important tool, which can easily generate high pressure by compressing small samples between two opposing diamond culets. However, the lack of temperature measurement accuracy is an ongoing problem [25]. Resistively heated diamond anvil cell (DAC) [27] controls the temperature by mounting an electrical heater with adjustable voltage and current out of the DAC body. In this case, the temperature inside DAC body can be easily controlled and measured. 

## 2. Materials and Methods 

The first group of tests was conducted on samples prepared under high temperature via an arc-melter at a low pressure. Mo_15_Nb_20_Re_15_Ta_30_W_20_ (master alloy), which was reported to have high hardness [28], and master alloy with dilute C-doped at 0.1 at. % (C_0.1_-alloy) samples were synthesized using the arc melting method (Edmund Bühler /MAM-1) under an argon atmosphere. C, Mo, Nb, Re, Ta, and W powders with purity of more than 99.5 % were mixed inside a polystyrene mill jar for 15 min and then pressed in a uniaxial die at 350 MPa pressure. To ensure chemical homogeneity, the melted ingots were inverted and remelted four times under the arc. To melt the samples, an arc melter was used under an argon atmosphere. As metal powders can be easily ejected from the arc region, metal powders were first mixed and then pressed in a uniaxial stainless-steel die to form disk shaped blocks. After loading the disk-shaped samples into the arc-melter processing chamber, the arc-melter vacuum system would pull vacuum and then high purity argon gas would refill the chamber. Typically, the evacuation/refill process was repeated at least three times to ensure a low residual oxygen level. With a proper chamber argon pressure (~0.3 bar), the arc was initiated to melt the sample on a water-cooled copper plate (no crucible was used). After complete melting, the arc was kept on for about 10 s. Then, the melted sample solidifies to form a button shaped ingot. This ingot would be flipped over, and the arc melting process was repeated for three more times. The repeated melting would ensure the chemical homogeneity within the ingots. Due to the repeated evacuation/refill process and the use of high purity Ar gas as the protective gas, the oxidation is kept to a minimum and thus not considered in this study. The crystal structure, microstructure, and hardness of C_0.1_-alloy and master alloy samples (synthesized using the same arc melting method) were examined to investigate the effects of C doping to the structural and mechanical properties of the alloy. The crystal structure of both types of samples were analyzed by a PANalytical Empyrean x-ray diffraction (XRD) system with 2 theta scans range from 20-120 degrees. The wavelength of the incident x-ray was 1.5406 Å. The microstructures of both types of samples were studied using scanning electron microscopy (SEM), and the chemical compositions were analyzed. The hardness characterization was performed with a SUN-TEC CM-802AT (V/K) hardness-testing machine. Five indents were conducted for each testing load to ensure the repeatability of the results. The space between adjacent indents was over three times the indent diagonal length. 

Further studies on performance of C_0.1_-alloy sample under high-temperature and high-pressure were performed by using the resister heated synchrotron DAC technique. The DAC XRD data under high-pressure and high-temperature were collected at beamline 12.2.2 at the Advanced Light Source (ALS), Lawrence Berkeley National Laboratory (LBNL) with an x-ray wavelength of 0.4959 Å. CeO_2_ was used as the standard material to calibrate the sample-detector distance and detector orientation and Au powder was used for the pressure calibration. The testing powders were loaded into the resister heated DAC with pressure-generating membrane. The pressure was controlled by pressure-generating membrane, and the temperature was controlled by an electrical heater with adjustable voltage and current, which was mounted out of DAC body. The DAC main body was cooled by a circulating liquid. The performance of C_0.1_-alloy under the combined high-pressure and high-temperature condition was studied by the high-pressure and high-temperature synchrotron XRD experiment. C_0.1_-alloy mixed with Au sample was firstly compressed at room temperature until 6 GPa, then was heated from room temperature to ~1000 °C in 10 h while pressure kept at 6 GPa. In this case, the performance of C_0.1_-alloy under high-pressure low-temperature (high P/low T) and under high-pressure high- temperature (high P/high T) could be investigated [29]. At last, the sample was cooled to room temperature in 2 h. The diffraction images at room temperature were initially analyzed by Dioptas [30] to determine the pressure by fitting to the standard Au diffraction peaks. The diffraction images under the combined high-pressure and high-temperature were refined to remove the background noise by material analysis [31] using diffraction (MAUD) software.

## 3. Results

### 3.1. Phase and Microstructure at Low-Pressure

**Figure 1:**
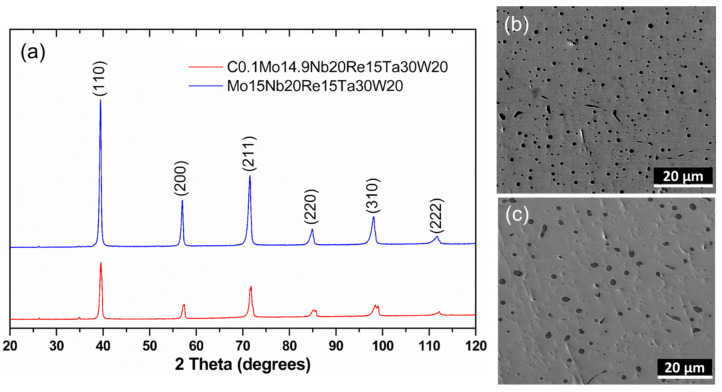
(**a**) XRD pattern of master alloy and C_0.1_-alloy, and SEM images showing microstructures of (**b**) master alloy and (**c**) C_0.1_-alloy.

**Figure 2:**
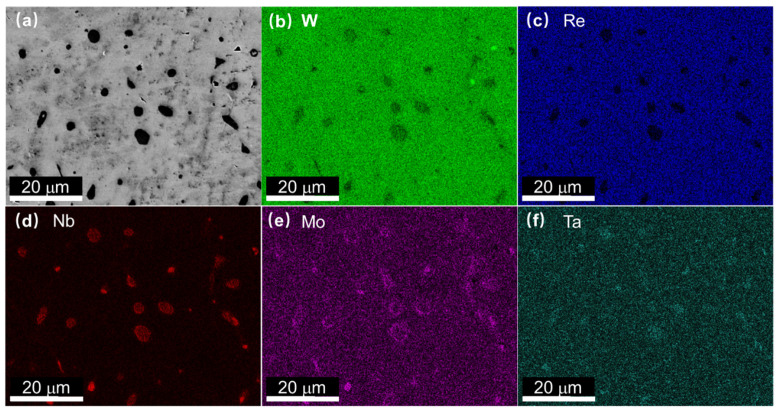
(**a**) SEM image of C_0.1_-alloy, energy-dispersive spectroscopy (EDS) mapping images for (**b**) W, (**c**) Re, (**d**) Nb, (**e**) Mo, and (**f**) Ta.

The XRD patterns of both master alloy and C_0.1_-alloy are shown in Figure 1a. As shown in the figure, only body center cubic (BCC) peaks were detected in both samples, indicating stable single BCC crystals. No carbide was obtained in C_0.1_-alloy under ambient pressure and temperature (low P/low T) based on the XRD observation. Considering that samples were already melted several times during synthesis but no XRD-peaks-related carbide were obtained, it is concluded that carbide could not be formed under ambient pressure and high temperature (low P/high T). Moreover, the peaks with the same crystallographic plane were in the plot, which is due to the almost identical lattice constants of both crystals, apart from the peaks at planes (220) and (310). The small differences in planes (220) and (310) are likely caused by the diffraction features of the segregations from the matrix in Figure 2. The doped C elements would not induce any noticeable changes on the lattice constant. According to Bragg’s law [32], the lattice parameters of master alloy and C_0.1_-alloy were both determined to be 3.210 Å. Because of the low concentration and small atom size of C, the effect from doped C to lattice constant of HEA is negligible. Similar observations on HEA such as Mo_0.5_NbHf_0.5_ZrTi [20] and CoCrFeMnNi [15] were reported.

SEM microstructure of master alloy and C_0.1_-alloy are shown in Figure 1b,c, respectively. Black dots in the SEM microstructures of the samples show the existence of segregations. No obvious morphological changes were observed in the SEM images due to the addition of low concentration of C. SEM second electron images and SEM energy-dispersive spectroscopy (EDS) elemental mapping of C_0.1_-alloy are shown in Figure 2 to demonstrate the chemical compositions of alloys. C element could not be identified using XRD/SEM techniques because of its low concentration. It has been identified that all regions in the SEM images are the BCC phase, which confirmed the analysis on XRD patterns that no carbide formed in C_0.1_-alloy. The EDS mapping images show that the dark areas were enriched in Nb, Mo, and Ta elements but depleted in W and Re. 

Based on the EDS composition analysis of C_0.1_-alloy in 9 selected areas, the average chemical compositions of C, Mo, Nb, Re, Ta, and W are listed in Table 1. As the reference, the chemical compositions in master alloy are also listed. The compositions of elements in both samples are very close. However, the Vickers micro hardness for both samples, which were tested at five different spots on the sample, showed a noticeable difference, as listed in the end of Table 1. The hardness values of both samples were obtained by using three different loads (gf)—100, 500, and 2000, respectively. It is clearly shown that Vickers hardness of master alloy reduced with the increase of load, while hardness of C_0.1_-alloy kept about the same under different loads. This indicates that the interstitial C atoms in the material help to pin the dislocations, and to restrain the movement of those dislocations. Several methods have been reported to calculate the Vickers hardness of alloys [28,33]. While the concentration of C in C_0.1_-alloy is too small to calculate by DFT, the role of mixture was employed to estimate the influence of C to the master alloy. The estimated Vickers hardness of master alloy is 8.33 GPa, and Vickers hardness of C_0.1_-alloy is 8.27 GPa. Both experimental observation and theoretical estimation show that influence of low concentration C to the hardness of alloys is very small.

### 3.2. C_0.1_-Alloy under High P/low T and High P/high T

**Figure 3:**
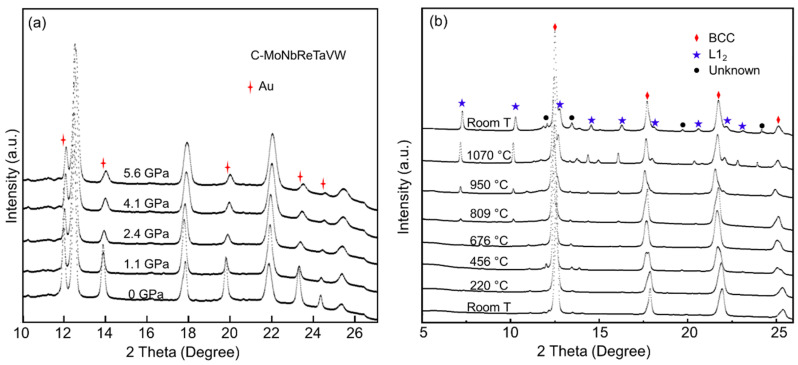
(**a**) XRD patterns of C_0.1_-alloy/Au mixture when pressure increased from 0 GPa to ~6 GPa. The corresponding pressures are marked on the top of each XRD pattern. XRD patterns of Au are marked by the red stars. (**b**) XRD patterns of C_0.1_-alloy/Au mixture with various temperatures when the pressure was 6 GPa. The corresponding temperatures are marked on the top of each XRD pattern. XRD patterns of body center cubic (BCC) phase are marked by red diamond; L1_2_ phase, blue star; unknown, black dots.

**Figure 4:**
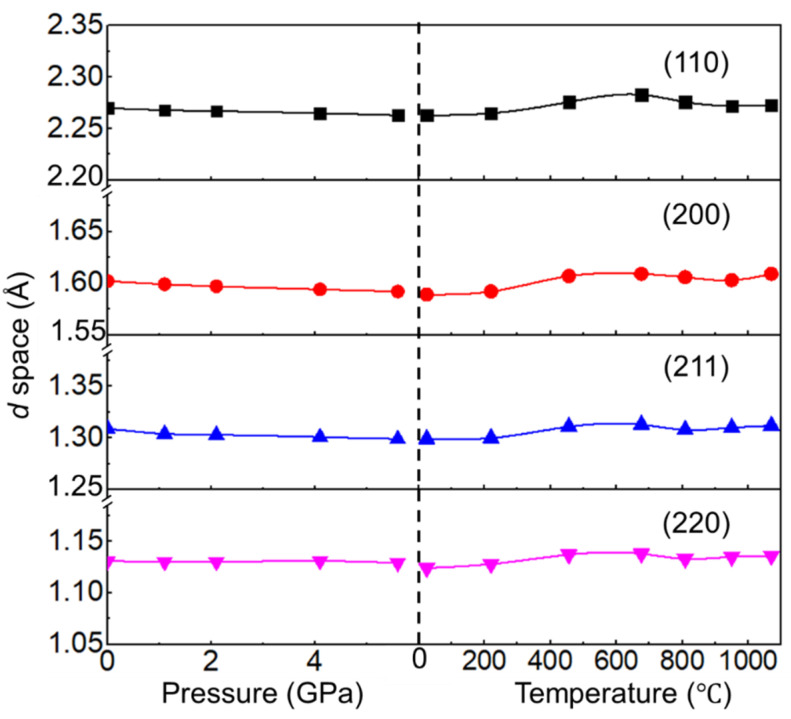
*d*-space as the function of pressure at room temperature (**left**) and *d*-space as the function of temperature at 6 GPa (**right**).

In situ XRD patterns of the C_0.1_-alloy/Au mixtures from low P/low T to high P/low T condition are shown in Figure 3a, where the XRD peaks of Au are marked. At room temperature and no applied pressure, C_0.1_-alloy was identified to have only a BCC phase with a lattice constant of 3.211 Å by DAC XRD, which is consistent with the observation of PANalytical Empyrean XRD. No new peak appeared during compression, indicating the stability of the BCC phase under a pressure up to ~6 GPa. No carbide was obtained under high P/low T condition. It is also clearly shown that all diffraction peaks shift to larger 2*θ* angles with increasing pressure. The corresponding *d*-spaces for (111), (200), (211), and (220) planes of C_0.1_-alloy as the functions of pressure at room temperature are shown in the left panel of Figure 4. *d*-spaces decrease as increasing pressure, indicting decreasing of lattice parameter. At 6 GPa, *d*-spaces of C_0.1_-alloy are about 0.5% smaller than that at 0 GPa.

After the sample was compressed to 6 GPa, the temperature was increased by applying the heater mounted out of DAC body. XRD patterns of C_0.1_-alloy/Au mixture from high P/low T to high P/high T are shown in Figure 3b. With the increase of temperature, atomic vibration increased, which lead to the increase of lattice constant. Consequently, the XRD peaks shift to the smaller angles while temperature increases. C_0.1_-alloy was found to have only BCC phase below 676 °C. A group of nonignorable peaks marked as blue star in Figure 3b appeared when the temperature increased to 809 °C, representing the formation of new phase. This new phase was identified to be the L1_2_ phase by QualX 2.0 [34], similar XRD peak of carbides with L1_2_ phase in AlCoCrFeNi was reported in Ref. [35]. Intensity of these new peaks became stronger when the temperature increased to above 1000 °C, indicating higher concentration of L1_2_ phase with increasing temperature. The new peaks existed even when samples were cooled back to room temperature, which indicated nonreversible phase transition happened in BCC C_0.1_-alloy under high pressure.

The *d*-spaces for (111), (200), (211), and (220) planes of C_0.1_-alloy as the functions of temperature with pressure kept at 6 GPa are shown in the right panel of Figure 4. *d*-spaces increase as temperature increases to 676 °C. The drop of *d*-space for all BCC planes was found when the temperature increased from 676 °C to 809 °C, indicating the formation of new phase in this temperature range. The phase transition leads to change of elements configuration in BCC phase. As a consequence, over the temperature range of 676 °C to 809 °C, the d-spaces of BCC crystal were smaller than those when the temperature was 676 °C. After 809 °C, *d*-spaces increased as temperature increased to 1070 °C. 

Combining the synthesis and characterization process, C_0.1_-alloy was treated through low P/high T, low P/low T, high P/low T, and high P/high T. Carbides were obtained under high P/high T and preserved back to the ambient temperature and pressure. The performance indicates that applied pressure and temperature could promote the formation of carbides when the doped concertation is low.

## 4. Discussion

To analyze the component of L1_2_ phase, the electronegativity difference (*∆^χ^_r_*) and chemical enthalpy of mixing *(∆H*_mix_) of binary equiatomic alloys according to Miedema’s model [36] between elements in C_0.1_-alloy were compared in Table 2. C-Ta pair has largest electronegativity difference, indicting strongest attractive interaction between C and Ta to form binary compounds. Moreover, electronegativity differences of C-Nb and C-Re are also relatively large compared with other pairs, which shows that C-Nb and C-Re binary compounds are also easily formed. The chemical mixing enthalpy listed in Table 2b shows that the pairs that contain C elements are much more negative than others, which indicates that binary intermetallic phase between C and other elements may easily form. Strongest binding strength exists between C and Nb, followed by Re, Mo, Ta, and W. 

L1_2_ phase is FCC phase with two elements (chemical formula: AB_3_) in one primitive unit cell. The space group of both L1_2_ phases is pm3m(221) with Pearson symbol of cP4. The database FactSage [37] listed the phase diagram of C-based binary alloy phase diagram at zero pressure. According to the database, C-Ta and C-Nb could form FCC phase, C-Mo and C-Re could form HCP phase, and C-W could form BCC phase. By combining the electronegativity difference and chemical mixing enthalpy analysis in Table 2, the L1_2_ phase identified under high-pressure and high-temperature is likely to be Ta_3_C and Nb_3_C binary compounds. TaC and NbC binary compounds were reported to have unique combination of properties, such as high-melting temperature, high hardness, good high-temperature strength, and good conductivity [38,39,40]. These carbides are usually employed as high-temperature structural materials in the form of hard constitutes in metal matrix composites.

## 5. Conclusions

In this work, the effect of dilute C-doped with a concertation of 0.1 at.% in RHEA master alloy was studied under both ambient pressure and high-pressure high-temperature conditions. Under low pressure, the XRD of RHEA with/without C shows the formation of pure BCC phase and lattice constant of 3.210 Å, indicating that the low concertation of C has an ignorable effect on the alloy phase and the crystal structure of RHEA. However, the doping of C has noticeable a impact on the hardness values of the RHEA. At a high pressure of 6 GPa, Ta_3_C and Nb_3_C Carbide with FCC_L1_2_ phase appear when the temperature is above 809 °C. The carbides of Ta_3_C and Nb_3_C are very stable even when temperature increased to above 1000 °C. As carbides are usually shown and favorable in high-temperature structural materials in the form of hard constitutes in metal matrix composites, the mechanical properties of carbon doped master alloy RHEA might be improved after a combined high-pressure and high-temperature process.

## Figures and Tables

**Table 1:** The chemical compositions (at.%) of master alloy (alloy) and C_0.1_-alloy (C-doped) and the Vickers micro hardness of both samples.

Sample	C	Mo	Nb	Re	Ta	W	Hardness		
							100 gf	500 gf	2000 gf
alloy	-	14.43 ± 0.17	22.74 ± 0.38	15.29 ± 0.09	26.66 ± 0.19	20.90 ± 0.19	6.451 ± 0.140	6.035 ± 0.303	5.400 ± 0.213
C-doped	0.06	14.32 ± 0.41	22.63 ± 0.54	18.12 ± 0.28	26.44 ± 0.32	18.43 ± 0.26	5.826 ± 0.104	5.905 ± 0.086	5.749 ± 0.203

**Table 2:** (**a**) The electronegativity difference *∆^χ^_r_* (by Pauling scale) and (**b**) values of chemical mixing enthalpy *∆H_mix_* (kJ/mol) of atomic pairs between elements for alloy C_0.1_-alloy.

	(a)	C	Mo	Nb	Re	Ta	W	Elements	
(b)									
C		-	−67	−102	−101	−60	−42	C	
Mo		0.39	-	−6	−7	−5	0	Mo	
Nb		0.95	0.56	-	−26	0	−8	Nb	
Re		0.65	0.26	0.3	-	−24	−4	Re	
Ta		1.05	0.66	0.1	0.4	-	−7	Ta	
W		0.19	0.2	0.76	0.46	0.86	-	W	
**Elements**		**C**	**Mo**	**Nb**	**Re**	**Ta**	**W**		**(a)**
								**(b)**	
